# Policy: New Environment Law for Afghanistan

**DOI:** 10.1289/ehp.114-a152

**Published:** 2006-03

**Authors:** David A. Taylor

In April 2005, Afghan president Hamid Karzai established the National Environmental Protection Agency (NEPA), the country’s first such entity. The event was strictly ceremonial, since Afghanistan still had no legal tool for environmental management. Eight months later, however, on 18 December 2005, the Afghan cabinet approved legislation that for the first time gives Afghanistan the legal power it needs to begin bettering its environment.

Known as the Environment Act, the law clarifies administrative roles at the national level and coordination with provincial authorities. It spells out frameworks for managing natural resource conservation and biodiversity, drinking water, pollution control, and environmental education. Equally as important, say its supporters, the law provides tools for enforcement.

“It seems to be a pretty sensible act,” says David Hanrahan, lead environmental specialist for South Asia at the World Bank. For example, the law’s environmental impact assessment process was guided by a review of 10 countries’ experience and vetted by environmental law experts at the World Bank, the World Conservation Union, and the UN, as well as by Afghan groups.

NEPA proposed the legislation based on recommendations issued in 2003 by a team of experts from the UN Environment Programme (UNEP). The UNEP findings were alarming: after two decades of conflict and drought, Afghanistan had lost nearly all of its wetlands and much of its forests, and its citizens were increasingly at risk for infections and epidemics caused by poor waste management and unequal access to fresh water. [For more on the UNEP findings, see “Environmental Triage in Afghanistan,” *EHP* 111:A470–A473 (2003).]

For two years, Asif Ali Zaidi, UNEP’s program manager in Afghanistan, has worked to help the government respond. UNEP facilitated consultations on the draft legislation with various agencies, citizen groups, and international officials, and funded translations of the draft law into Dari and Pashto, the country’s official languages. Besides NEPA (which emerged from the former Ministry of Irrigation, Water Resources, and Environment), other agencies with key roles under the new law include the Ministry of Justice and the Ministry of Agriculture, Animal Husbandry, and Food.

In Afghanistan, more than 80% of the population relies directly on natural resources such as rangelands and water bodies for their livelihood and daily needs, and only 12% of the land is arable. Thus, widespread environmental degradation poses a threat to livelihoods and places the poorest Afghans at particular risk, wrote Zaidi and Belinda Bowling, UNEP’s environmental law expert in Afghanistan, in the Fall 2005 issue of *Sustainable Development Law & Policy*.

Zaidi and Bowling linked environmental issues directly with Afghans’ top concern, security, observing that environmental degradation in Afghanistan—often the consequence of socioeconomic inequities—is a factor contributing to prevalent insecurity. In one example, desperate subsistence farmers displaced by desertification and crop failure are more likely to cultivate poppies as part of the drug trade, which is intrinsically linked to insecurity in Afghanistan.

Protecting natural resources in a country lacking basic infrastructure has posed a serious challenge. The National Development Framework, a 2002 map for Afghanistan’s economic development, did not mention the environment, although Zaidi says it was understood as “an important cross-cutting issue.” In 2004, another approach emerged in the Afghanistan National Development Strategy, an Afghan-specific version of the UN Millennium Development Goals couched as a five-year plan. These overlapping schemes have caused confusion among planners, compounded by a lack of baseline data on forest cover, energy use, and other indicators.

According to Bowling, the new law will help spur institutional reform and the development of regimens for pollution control and environmental impact assessments, among other things. “Like most fledgling institutions,” she says, “NEPA now requires time to establish itself properly within the new government structure.” UNEP plans to support much of the reform through its Post-Conflict Branch.

Hanrahan is cautiously optimistic about the recent developments. Most Afghans, he says, care about their environment. He notes the people have a history of cooperative practices, for example in ancient irrigation systems, adding, “This is where civilization comes from.”

## Figures and Tables

**Figure:**
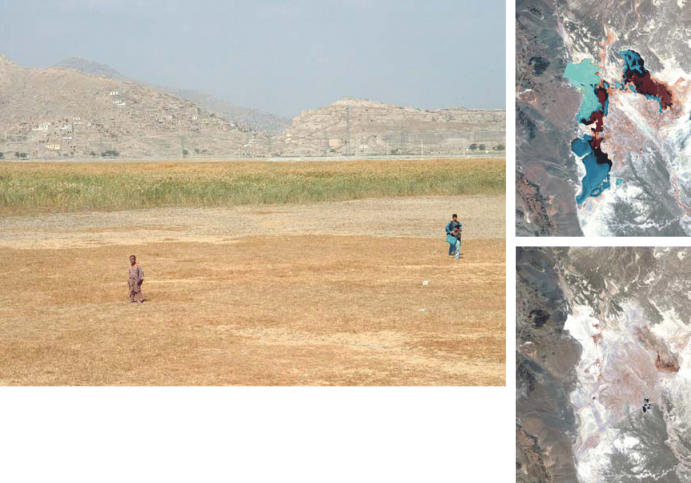
Thirsting for reform. The extinct Hamoun wetlands (top right in 1976, while still thriving; bottom right in 2001, as a desiccated salt flat) and Kole Hashmat Khan wetlands (above) are just two illustrations of how years of drought and conflict have stripped Afghanistan of its natural resources. A new agency and guiding law offer hope that life can be breathed back into Afghanistan’s environment.

